# Is left-behind status related to differences in sexual health of Armenian mothers? Evidence from the Demographic and Health Survey in 2010 and 2015

**DOI:** 10.1371/journal.pone.0228344

**Published:** 2020-02-03

**Authors:** David Ferrandiz-Mont, Chi Chiao

**Affiliations:** 1 Institute of Public Health, International Health Program, School of Medicine, National Yang-Ming University, Taipei, Taiwan; 2 Institute of Health and Welfare Policy, Institute of Public Health, School of Medicine, National Yang-Ming University, Taipei, Taiwan; University of Cape Coast, GHANA

## Abstract

**Background:**

Migration caused by poverty is a growing public health issue around the world. Migrants are at heightened risk of HIV/STIs and yet the vulnerability to poor sexual health of their left-behind partners, in relation to their household wealth, remain understudied. This investigation examines differences in sexual health from 2010 to 2015 among Armenian mothers, with a specific focus on their left-behind migration status and household wealth.

**Methods and findings:**

Using the population-based Demographic and Health Surveys from Armenia, multilevel logistic models were used to examine the various relationships between sexual health, left-behind status, and household wealth. The multivariate analysis results showed that self-reported sexually transmitted infection (STI) symptoms (AOR = 1.45; *p*<0.01) and intimate partner violence (IPV) (AOR = 1.45; *p*<0.01) increased from 2010 to 2015; furthermore, negotiation power over sex (AOR = 0.77; *p*<0.01) declined among Armenian mothers. Left-behind mothers (LBMs) were more likely to report STI symptoms than their non-LBM counterparts (AOR = 1.61; *p*<0.01). In addition, significant differences in sexual health between LBMs and non-LBMs with different levels of household wealth were observed. The poorest wealth quintiles were associated with a higher likelihood of self-reported STI symptoms (AOR = 1.74; *p*<0.05) and IPV (AOR = 1.78; *p*<0.01), as well as a lower likelihood of utilizing HIV testing (AOR = 0.48; *p*<0.01) and negotiating power over sex (AOR = 0.47; *p*<0.01).

**Conclusions:**

This study strives to fill gaps in the literature related to the relationship between left-behind status, household wealth, and sexual health among Armenian mothers in a context of economic expansion. Among these mothers, poor sexual health outcomes increased from 2010 to 2015. Both low household wealth and a left-behind status were associated with adverse sexual health outcomes. These findings suggest future campaigns aimed at improving the sexual health of Armenian mothers need to be migration-status appropriate and socioeconomic-sensitive.

## Introduction

Given the fast-increase in migration throughout the world [1], a growing body of literature has attempted to bring policymakers attention to the crucial role of migration in shaping the epidemiology of infectious diseases. Sexual health is severely affected by migration dynamics as has been highlighted by several studies targeting the risk of HIV/STI infection among labor migrants. Male migrants tend to engage in a higher level of HIV/STI-related risky behaviors and this results in an increased risk of contracting HIV/STIs while in their host country [2, 3]. It is important to noted that migrants not only have a heightened risk for HIV/STI infection compare to non-migrants, but that they also serve as a bridging population and transmit those infections to others, including their left-behind partners in their home country [4]. The literature shows that the prevalence of HIV/STIs among left-behind female partners is also on the rise, and some researchers have hypothesized that male migrants are likely to be the bridge by which HIV/STIs move from the host country to the home country and infect left-behind sexual partners [5–8]. In addition, the intimate context in which these left-behind partners live may further lead to a particularly vulnerable situation, including a higher likelihood of intimate partner violence (IPV), a lower likelihood of negotiating power over sex and a lower ability to utilize a HIV testing service.

Based on the above, left-behind female partners are likely to be more exposed to coercive control by their male partners than their non-left-behind partners, particularly in a patriarchal society. Owing to the long-distance nature of the relationship between partners, migrant male partners may adopt complicated strategies within their households to “control” their left-behind female partners. For example, the male partner's relatives may exert control over them [9] or, quite commonly, they may limit the ability of their spouses to acquire and utilize economic resources [10]; the latter is considered to be economic abuse of their partner [11]. In societies with more traditional gender norms, such as Guatemala or Armenia, left-behind wives are frequently forced to leave their job and focus on being a good mother and wife within the household [10]. This unemployed situation limits women to the household sphere and increases their dependence on their husband’s income, which in turn places these left-behind wives in a vulnerable position. These vulnerabilities, in terms of sexual health include an increased risk of exposure to IPV, a reduction in negotiation power regarding sex, and reduced likelihood of utilizing HIV testing; these when taken together increase the risk of acquiring STIs [12, 13]. In addition to this, motherhood can also accentuate the vulnerability of left-behind mothers (LBM) in terms of poor sexual health outcomes. Mothers often will have an even higher economic dependence on their male partner's as they have to provide for their children as well as themselves. This situation forces them to stay longer with their violent male partners for the sake of their offspring [14], which increases the severity of intimate partner violence [15] as well as the risk of acquiring an STI, including HIV [16].

Thus, the population of LBMs seems to be particularly vulnerable to adverse sexual health outcomes and socioeconomic characteristics; furthermore, these factors are likely to play a crucial role in their sexual health. Household economic deprivation represents a major barrier to accessing health services [17] and is a risk factor for IPV [18, 19]. The main drivers for migration in Central and Eastern European countries are related to the financial standing of the family [20, 21] and thus the vast majority of LBMs are from low socioeconomic group families [22]. As a result, the association between household socioeconomic characteristics and sexual health among LBMs can be hypothesized contribute to status of the left-behind partners.

Accordingly, household wealth and left-behind status may take a toll on the sexual health of mothers, particularly in less developed countries with a patriarchal society and a high rate of male migration, one such example being Armenia. Migration is a common phenomenon in Armenia, where the economic situation has compelled a large number of citizens to work abroad. Armenian migrants residing abroad represented around 20% of the total population in 2012, with the majority of them being males. This has resulted in a large number of female partners being left behind [23]. Moreover, migration seems to have had a significant influence on the epidemiology of sexually transmitted infection, including HIV. From 2000 onwards, the number of women infected with HIV has increased substantially. In 2000 the number of new female HIV positive cases was only 6, but in 2014 this number had reached 117 cases. Interestingly, the majority of the HIV positive female cases that were detected between 2011 and 2015 were partners of migrants, which indicates that migration has become a major cause of the HIV spread in Armenia [24]. It is noteworthy that over the last decade, Armenian society has experienced remarkable economic changes [25] and that these seems to have shaped the sexual vulnerability of LBMs. These economic changes were aimed at reducing poverty, but have also fueled economic inequalities, while, over the same period, the level of women’s empowerment in the labor marketplace has remained unchanged [26, 27].

The present study aims to fill gaps in the literature related to the relationship between left-behind status, household wealth, and sexual health among Armenian mothers in a context of economic expansion and social change. Due to the increase in economic disparities and the presence of persistent gender inequalities in Armenian over the period 2010 to 2015, our first objective was to examine the differences in sexual health outcomes in 2010 and 2015. Secondly, we have explored whether or not a left-behind status is able to explain these sexual health disparities to any meaningful extent. Thirdly, the present investigation has in particular focused on the differences in sexual health outcomes between LBMs and non-LBMs in 2010 and 2015 while taking their household SES into consideration.

## Methods

### Data and study population

The present study employed two nationally-representative household surveys, specifically the Armenia Demographic and Health surveys (ADHS) that took place in 2010 and 2015. These surveys provide a wide range of information, including factors related to migration, such as the husband’s migration status, and socioeconomic characteristics, including household wealth, employment status or education, as well as sexual health-related outcomes, including HIV testing utilization, self-reported STI symptoms, negotiating power over sex or intimate partner violence. The ADHS data was collected using a two-stage sampling strategy and was obtained for this study from the publicly available MEASURE DHS website: www.measuredhs.com. Further details about the data collection and sampling design are described in the survey reports [28, 29].

Data collection and analysis were performed under strict ethical standards. The ADHS procedure has been approved by the ethical review boards of Macro International Inc. (a U.S.-based company that provides technical assistance to DHS surveys worldwide), various ethical review boards in the host country, and other relevant implementing partners. In DHS surveys written informed consent was obtained at the start of the interviews in all cases. Furthermore, all data was anonymized before use. The study protocol for the present study was reviewed and approved by the Institutional Review Board (IRB) of National Yang-Ming University in Taiwan (IRB number YM107046E).

The present study focused on Armenian women of reproductive age (15 to 49 years old) who were pregnant or had children. After discarding respondents with incomplete data, 8,025 mothers where selected. These consisted of 3,874 mothers from 308 communities in the 2010 dataset and 4,151 mothers from 313 communities in the 2015 dataset. Among these mothers, 1,633 women were classified as left-behind mothers and the rest as non-left-behind mothers.

### Outcome measures

The sexual health outcomes used in this study consisted of (1) self-reported STI symptoms, (2) HIV testing service utilization, (3) IPV, and (4) negotiating power over sex. All outcomes were assessed based on the women’s reports as collected by the ADHS. Self-reported STI symptoms were considered positive if the respondent reported the presence of a genital sore/ulcer and/or a genital discharge during the last 12 months prior to the interview (“positive” coded as 1; “otherwise” coded as 0). HIV testing was assessed by whether or not women had ever been tested for HIV within the last 23 months prior the interview (“yes” coded as 1; “otherwise” coded as 0). IPV was assessed by whether women agreed that a male partner is justified in beating his partner in at least one of the following situations: “the wife goes out without telling her husband”, “the wife neglects her children”, “the wife argues with her husband”, “the wife refuses to have sex with her husband” and “the wife burns the food” (“yes” coded as 1; “otherwise” coded as 0). Participant women were considered to have negotiation power over sex if she reported that they can refuse to have sex with her male partner.

### Explanatory variables

The main explanatory variables of this study were left-behind status, household wealth and the year of the survey. Left-behind status was assigned to those mothers whose male partners had been working abroad for three months or longer in the past three years. The household wealth index consists of asset-based measurements [30] that were analyzed into five quintiles; these were ranked as poorest, poorer, middle, richer and richest. Time is an important indicator in this study because prior research in this area has been mostly cross-sectional. The present investigation seeks to explore differences in sexual health through the use of two population-based surveys. Datasets from 2010 and from 2015 were utilized and a categorical variable was created to distinguish the data collected at these two time points.

In addition, this study also included socio-demographic characteristics, namely the age of the mother, her employment status, her education attainment, her knowledge of HIV, the number of children under five years old in her household, and the region and residence where they were settled; these were all used as covariates. The age of the mother is divided in the following categories: 15–29, 30–39, and 40–49 years old; education attainment is an ordinal variable, with a range from no education (coded as 0) to higher than secondary education (coded as 5). Current employment status is a dichotomous variable (employed and another state); the number of children under five is a continuous variable, with a possible range from 0 to 5. Knowledge of HIV was assessed based on the definition by UNICEF of comprehensive HIV knowledge [31]. Mothers were considered to have a comprehensive knowledge of HIV/AIDS if they met the following three criteria: (1) they can identify the following two methods to reduce HIV transmission: condom use and having sex with only one partner who has no other sex partners; (2) they are able to acknowledge that a healthy-looking person may have HIV/AIDS; and (3) they can identify two of the most common misconceptions about HIV, such as “AIDS virus can be transmitted by mosquito bites” and “a person can become infected by sharing food with a person who has the AIDS virus”. The variable that reflected the geographic region of the subject consists of eleven categories or geographic areas. Using this variable, regions were grouped into two categories depending on the percentage of LBMs living in each region; if the region had more than 20% LBMs in the pooled data, the category was labeled as “a region with a higher migration rates”, namely Armavir, Gegharkunik, Lori, Kotayk and Shirak. If the region had 20% LBMs or less in the pooled data, it was named “a region with a lower migration rates”, namely Aragatsotn, Ararat, Syunik, Vayots Dzor, Tavush, and Yerevan. This categorization is consistent with reports about migration rates in Armenia [20, 32]. Finally, residency was dichotomized into either “urban” or “rural”.

### Statistical analysis

Data analysis was performed using Stata 14 and all analyses were weighted to adjust for the sample design. We began with bivariate analysis that characterized the disparities in sexual health between 2010 and 2015 among Armenian mothers, and during this process we analyzed LBMs and non-LBMs separately. Mothers within a community often experience common community level influences and their sexual health patterns thus may be more similar than those of individuals across communities. In order to estimate the significance of such community influences, we calculated the percentage of the total variance related to experiencing the studied adverse sexual health outcomes that was related to the community, namely the intraclass correlation coefficients (ICCs) or the intracommunity correlation [33]. The fact that the ICC values showed significant variation indicates that this would seem to be explained by community-level variables. Accordingly, we conducted a two-level multilevel regression where the mothers were at level 1, which was nested within level 2, namely the communities. We estimated the ICCs using a two-level multilevel logistic regression model without explanatory variables, namely a null model, for each of sexual health outcomes.

To assess the research objectives, the analytical strategy of the present study was conducted using progressive strategies. The covariates included in the regression models were selected using the likelihood ratio test (LRT). We began with a model that consisted only of the main exposure variables (left-behind status, household wealth, and year of the survey). Then, we included the various covariates progressively, assessing whether their inclusion significantly improved the goodness of fit of the model. Model 1 attempts to determine the independent influences of left-behind status, household wealth and year of the survey on the subjects' sexual health outcomes. Given that the present study also explored the contribution of left-behind status and household wealth to sexual health differences over the same study period, Model 2 adds interactions terms that examine how the year of the survey affects the association between left-behind status and household wealth for each of sexual health outcomes. Moreover, multilevel logistic models, stratified by left-behind status, were performed to examine the influence of household wealth on the sexual health of Armenian mothers.

## Results

Table 1 shows the distribution of the Armenian mothers' characteristics by survey year and whether or not they were LBMs. Across two survey years, LBMs were more likely than non-LBMs to have a lower level of education attainment, lower HIV comprehensive knowledge, and to reside in rural areas. In 2010, a higher proportion of LBMs lived in the Armenian regions of Gegharkunik, Lori, and Shirak compared to non-LBMs, and, in addition to these regions, in 2015 the areas of Armavir and Kotayk also had a higher proportion of LBMs. The percentage of LBMs living in the mentioned regions together accounted for about 65% and 70% of the total number of LBMs in 2010 and 2015, respectively. In 2015 about a quarter (23.1%) of LBMs came from the lowest quintile of household wealth, compared to 17.2% of non-LBMs. In terms of sexual health outcomes, LBMs were more likely than non-LBMs to report STI symptoms in 2010 (4.2% *vs*. 2.6%) and in 2015 (8.0% *vs*. 4.4%), with the rate of self-reported symptoms increasing approximately twofold for both LBMs and non-LBMs between surveys. While IPV rates were similar for non-LBMs (10.7%) and LBMs (10.9%) in 2010, it was clear that the IPV rate had increased dramatically among LBMs in 2015 (16.1%). Similar percentages for LBMs and non-LBMs negotiating power over sex for 2010 (71.44% *vs*. 72.70%) and for 2015 (67.84% *vs*. 68.91%) were similar for each survey, although it should be noted that there were decreases that affected both groups. Less than 10% of non-LBMs and LBMs reported recent HIV testing in 2010 and 2015 surveys.

**Table 1:** Percentage distribution of sociodemographic characteristics and sexual health outcomes of mothers by left-behind status, Demographic and Health Surveys (DHS) 2010 and 2015 in Armenia.

	ADHS 2010			ADHS 2015		
	Non-LBM	LBM		Non-LBM	LBM	
	N = 3,078	N = 796	*p*-value	N = 3,314	N = 837	*p*-value
**Socio-demographic characteristics**						
Age (%)						
15–29 years old	29.24	24.12	*0*.*082*	25.80	29.34	*0*.*152*
30–39 years old	32.54	33.37		40.46	38.61	
40–49 years old	38.22	42.51		33.74	32.05	
Education attainment [mean (SD); range 0–5]	4.52 (0.59)	4.40 (0.62)	*< 0*.*001*	4.46 (0.65)	4.38 (0.62)	*< 0*.*01*
Number of children under five in the household [mean (SD); range 0–5]	0.56 (0.82)	0.51 (0.83)	*0*.*017*^†^	0.57 (0.77)	0.61 (0.83)	*0*.*346*^†^
Currently employed (%)	33.58	36.98	*0*.*236*	37.24	30.73	*< 0*.*01*
Comprehensive knowledge about HIV/AIDS (%)	25.68	21.21	*< 0*.*05*	31.15	24.00	*< 0*.*001*
Regions with higher migration rates (%)	42.36	64.83	*< 0*.*001*	37.98	69.66	*< 0*.*001*
Armavir	10.52	8.10	*< 0*.*001*	9.94	12.14	*< 0*.*001*
Gegharkunik	5.96	16.21		4.87	17.57	
Lori	7.84	13.13		4.61	8.67	
Kotayk	10.14	7.82		11.22	15.82	
Shirak	7.88	19.57		7.33	15.45	
Regions with lower migration rates (%)	57.64	35.17		62.02	30.34	
Aragatsotn	4.66	3.37		5.16	3.11	*< 0*.*001*
Ararat	6.54	5.38		10.84	4.09	
Syunik	4.46	0.26		5.08	0.48	
Vayots Dzor	2.45	2.46		2.27	1.35	
Tavush	4.54	3.58		5.02	5.32	
Yerevan	34.99	20.10		33.63	15.97	
Urban residency	62.10	47.75	*< 0*.*001*	61.33	44.85	*< 0*.*001*
Household wealth (%)							
Q1—lowest	19.94	21.91	*< 0*.*001*	17.22	23.13	*< 0*.*001*
Q2	19.41	26.59		19.71	24.84	
Q3	18.82	24.66		18.19	22.00	
Q4	19.72	14.58		20.71	16.50	
Q5—highest	22.11	12.25		24.16	13.53	
**Sexual health outcomes**						
Self-reported STI symptoms (%)	2.60	4.16	*< 0*.*05*	4.35	8.03	*< 0*.*001*
Intimate partner violence (%)	10.68	10.91	*0*.*881*	9.73	16.12	*< 0*.*001*
Negotiating power over sex (%)	72.70	71.44	*0*.*606*	68.91	67.84	*0*.*590*
Recent HIV testing (%)	7.42	6.12	*0*.*223*	8.54	6.87	*0*.*119*

Note: N is unweighted; percentages and means are weighted. Percentages may not sum to 100 owing to rounding.

^†^*p*-value is calculated using Somer’s D test.

The present study calculated ICC to verify if an analytical strategy involving multilevel logistic models is appropriate. The ICC for reporting STI symptoms is 0.21 in 2010 and 0.17 in 2015, which suggests that 21% of the total variation when reporting STI symptoms can be explained by the communities in 2010 and this was reduced to 17% in 2015 (*p*<0.01). Likewise, the ICCs of utilizing a HIV testing service were 0.16 in 2010 (*p*<0.01) and 0.17 in 2015 (*p*<0.01), of reporting IPV were 0.22 in 2010 and 0.47 in 2015, and of having negotiating power over sex were 0.07 in 2010 (*p*<0.01) and 0.09 in 2015 (*p*<0.01).

Table 2 presents the multilevel logistic regression results that estimate separately for each outcome the likelihoods of sexual health. Model 1 examines the independent associations, in terms of time interval, the left-behind status, the household wealth, and the sexual health outcomes of the surveyed Armenian mothers, adjusting for individual background characteristics. The primary interests regarding Model 1 are regarding the effects of year, left-behind status, and household wealth. For self-reported STI symptoms, the analyses show significant overall increases among Armenian mothers (AOR = 1.45; *p*<0.01), even after adjusting for the background variables. LBMs were more likely than non-LBMs to report STI symptoms (AOR = 1.61; *p*<0.01); Armenian mothers in the poorest household wealth quintile were more likely than those in the highest quintile to report STI symptoms (AOR = 1.74; *p*<0.05). Model 2 adds interaction terms for household wealth and survey year and tests for difference in the effects of household wealth over the 5-year period. This was able to identify an appreciable change in the significance of the odds of reporting STI symptoms between 2010 and 2015, indicating that the time differences in self-reported STI symptoms is dependent on household wealth. Thus, there was an interactive significant effect between household wealth and self-reported STI symptoms over time among Armenian mothers. For example, Armenian mothers in households with the poorest wealth quintile were found to have self-reported STI symptoms odds that were 1.06 times higher than Armenian mothers in households with a highest wealth quintile; this poorest wealth quintile household effect appeared to be particularly significant in 2015 (where the effect was 1.06 × 2.53, that is 2.7).

**Table 2:** Multilevel logistic regression models of the sexual health of Armenian mothers by left-behind status, 2010 and 2015.

		Self-reported STI Symptoms		Recent HIV Testing		Intimate Partner Violence		Negotiating Power over Sex	
		Model 1	Model 2	Model 1	Model 2	Model 1	Model 2	Model 1	Model 2
*Fixed effects*		OR (95% CI)	OR (95% CI)	OR (95% CI)	OR (95% CI)	OR (95% CI)	OR (95% CI)	OR (95% CI)	OR (95% CI)
Year (ref = 2010)									
	2015	1.45** (1.15–1.82)	0.66 (0.32–1.37)	1.08 (0.90–1.30)	1.38 (0.88–2.15)	1.45**(1.25–1.68)	1.23 (0.76–1.98)	0.77** (0.69–0.85)	0.79 (0.59–1.05)
Left-behind status (ref = No)									
	Yes	1.61** (1.25–2.07)	1.59**(1.23–2.05)	0.79^§^ (0.62–1.01)	0.78^§^ (0.62–1.00)	1.19^§^ (1.00–1.42)	1.19* (1.00–1.43)	1.05 (0.92–1.19)	1.05 (0.93–1.19)
Household wealth (ref = The 5th quintile, Q5: the highest)									
	The 1st quantile, Q1	1.74* (1.05–2.86)	1.06 (0.54–2.09)	0.48** (0.32–0.72)	0.52*(0.29–0.92)	1.78** (1.27–2.50)	2.07**(1.29–3.33)	0.47** (0.38–0.59)	0.47**(0.35–0.64)
	The 2nd quantile, Q2	1.49^§^ (0.93–2.40)	0.82 (0.42–1.58)	0.75^§^ (0.53–1.05)	0.99 (0.62–1.59)	1.50* (1.08–2.05)	1.38 (0.87–2.17)	0.54** (0.44–0.66)	0.51**(0.39–0.68)
	The 3rd quantile, Q3	1.78** (1.15–2.76)	1.20 (0.65–2.20)	0.74^§^ (0.54–1.01)	0.76 (0.48–1.21)	1.22 (0.90–1.66)	0.91 (0.57–1.43)	0.61** (0.50–0.73)	0.63**(0.48–0.83)
	The 4th quantile, Q4	1.23 (0.78–1.96)	0.87 (0.46–1.67)	0.87 (0.65–1.16)	1.08 (0.71–1.67)	1.44* (1.08–1.94)	1.17 (0.75–1.82)	0.73** (0.61–0.88)	0.82 (0.62–1.08)
	Q1 × 2015		2.53*(1.06–6.04)		0.88 (0.44–1.72)		0.78 (0.44–1.39)		1.03 (0.71–1.49)
	Q2 × 2015		2.97*(1.23–7.13)		0.58^§^(0.32–1.06)		1.17 (0.66–2.07)		1.13 (0.80–1.62)
	Q3 × 2015		2.13^§^ (0.91–4.98)		0.96 (0.53–1.72)		1.67^§^ (0.94–2.97)		0.93 (0.65–1.33)
	Q4 × 2015		1.92 (0.77–4.82)		0.67 (0.37–1.20)		1.44 (0.80–2.58)		0.81 (0.56–1.17)
**Socio-demographics**									
Age (ref = 15–29 years-old)									
	30–39	1.20 (0.87–1.66)	1.21 (0.87–1.68)	0.28** (0.22–0.35)	0.28**(0.22–0.35)	1.08 (0.88–1.34)	1.09 (0.88–1.34)	1.11 (0.95–1.27)	1.11 (0.96–1.28)
	40–49	1.13 (0.80–1.61)	1.14 (0.80–1.61)	0.12** (0.09–0.17)	0.12**(0.09–0.17)	1.13 (0.91–1.41)	1.13 (0.91–1.42)	0.97 (0.84–1.13)	0.97 (0.84–1.13)
Educational attainment		0.81* (0.67–0.97)	0.81* (0.67–0.97)	1.28** (1.08–1.51)	1.29** (1.08–1.51)	0.74**(0.65–0.84)	0.74**(0.65–0.83)	1.39** (1.28–1.51)	1.39**(1.28–1.52)
Currently employed (ref = Not employed)		1.11 (0.88–1.42)	1.13 (0.88–1.44)	0.98 (0.79–1.22)	0.98 (0.78–1.22)	0.98 (0.83–1.15)	0.98 (0.84–1.16)	0.79** (0.71–0.89)	0.79**(0.71–0.89)
Comprehensive knowledge about HIV/AIDS		0.67** (0.51–0.90)	0.67**(0.51–0.90)	1.25* (1.02–1.53)	1.25* (1.02–1.53)	0.61** (0.51–0.73)	0.61**(0.51–0.73)	1.65** (1.46–1.86)	1.65**(1.46–1.87)
Number of children under five in the household		0.88 (0.73–1.05)	0.88 (0.73–1.05)	1.47** (1.32–1.66)	1.47**(1.31–1.64)	1.03 (0.92–1.15)	1.03 (0.93–1.15)	1.04 (0.96–1.13)	1.04 (0.96–1.12)
		**Self-reported STI Symptoms**		**Recent HIV Testing**		**Intimate Partner Violence**		**Negotiating Power over Sex**	
		Model 1	Model 2	Model 1	Model 2	Model 1	Model 2	Model 1	Model 2
*Fixed effects*		OR (95% CI)	OR (95% CI)	OR (95% CI)	OR (95% CI)	OR (95% CI)	OR (95% CI)	OR (95% CI)	OR (95% CI)
*(Continued)*									
Region (ref = those with low migration rates)									
	With high migration rates	0.71* (0.53–0.94)	0.72* (0.54–096)	1.74** (1.37–2.20)	1.72**(1.36–2.18)	0.78^§^ (0.59–1.03)	0.78^§^ (0.59–1.04)	0.92 (0.81–1.05)	0.92 (0.81–1.05)
Urban residency		0.77 (0.56–1.05)	0.77 (0.56–1.07)	0.87 (0.66–1.14)	0.85 (0.65–1.12)	0.72** (0.56–0.92)	0.74*(0.58–0.95)	0.82* (0.71–0.95)	0.83*(0.72–0.97)
Comparison to previous model^‡^									
	Chi square		3.27		2.60		6.30		2.35
	Degrees of freedom		4		4		4		4

Notes

^§^ *p* < 0.10

*p < 0.05

**p < 0.01; *Abbreviations*: OR, odds ratio; CI, confidence interval.

^‡^We adjust interactions terms that examine whether the associations between houeshold wealth and sexual health outcomes depeneded on survey year for Model 2 and found these models basically revealed no appreciable differences from Model 2, and resulted in no significant improvement in fit over Model 2. As a result, these interaction terms were not included in Table 3.

Like the rise in self-reported STI symptoms over the studied period, IPV also increased between from 2010 to 2015 (AOR = 1.45; *p*<0.01); and a lower household wealth is also significantly associated with an increase odds of IPV. When mothers from the richest quintile are compared with mothers from the poorest wealth quintile (AOR = 1.78; *p*<0.01) and with the poorer wealth quintiles (AOR = 1.50; *p*<0.05), they were found to be more likely to report IPV in both the above cases. Nevertheless, the IPV increment was found not to be affected by household wealth. In contrast to the differences over time in self-reported STI symptoms and IPV, negotiation power over sex declined during the study period (AOR = 0.77; *p*<0.01); furthermore, the richer the household, the less likely was the subject to report having ability to negotiate sex. In addition, the non-significant interaction term in Model 2 showed that the effect of the year of the survey on having negotiating power over sex did not differ by household wealth. Turning to HIV testing, Armenian mothers from the poorest household wealth were less likely than those from the richest household wealth to utilize HIV testing (AOR = 0.48; *p*<0.01). Nevertheless, the effect of the year of the survey on HIV testing was found not to vary significantly by level of household wealth.

In order to investigate the effect of household wealth on sexual health outcomes between LBMs and non-LBMs, multilevel logistic regression models were conducted to estimate the adjusted odds of household wealth affecting sexual health among LBMs and non-LBMs; this analysis took the survey year, the age of the mother, the educational attainment of the mother, the employment status of the mother, the number of children under 5 years of age in the household, the HIV knowledge, and the region and residency were mothers lived into account (Table 3). Among non-LBMs, an increase in household wealth was found to be significantly associated with a decreased odds of self-reported STI symptoms and IPV, but with an increased odds of negotiating power over sex. The poorer the household, the lower were the odds of utilizing HIV testing, and, similar to non-LBM households, poorer LBM households were associated with a lower odds of utilizing HIV testing and a decreased odds of negotiating power over sex. In addition, a higher odds of IPV was associated with being a poorer household among the LBMs group. Finally, among LBMs no significant associations were observed between household wealth and self-reported STI symptoms.

**Table 3:** Association (OR, 95% CI) between household wealth and sexual health related outcomes by left-behind status, 2010 and 2015 data.

			Left-behind Armenian mothers N = 1,633								Non-left-behind Armenian mothers N = 6,392						
			Self-reported STI Symptoms		Recent HIV Testing		Intimate Partner Violence		Negotiating Power over Sex		Self-reported STI Symptoms	Recent HIV Testing		Intimate Partner Violence		Negotiating Power over Sex	
*Fixed effects*			OR (95% CI)		OR (95% CI)		OR (95% CI)		OR (95% CI)		OR (95% CI)	OR (95% CI)		OR (95% CI)		OR (95% CI)	
Household wealth (ref = The 5th quintile, Q5: the highest)																	
	The 1st quantile, Q1		1.17 (0.44–3.11)		0.34* (0.14–0.82)		2.08* (1.00–4.34)		0.55* (0.33–0.92)	2.00* (1.10–3.63)		0.50**(0.32–0.78)		2.00** (1.36–2.92)		0.45**(0.35–0.58)	
	The 2nd quantile, Q2		0.92 (0.35–2.37)		0.58 (0.27–1.27)		1.39 (0.68–2.83)		0.64^§^ (0.39–1.04)	1.78* (1.02–3.10)		0.75 (0.52–1.10)		1.71** (1.20–2.45)		0.51**(0.41–0.64)	
	The 3rd quantile, Q3		1.18 (0.49–2.87)		0.44* (0.22–0.93)		1.18 (0.60–2.31)		0.65^§^ (0.41–1.02)	2.07** (1.25–3.45)		0.81 (0.58–1.13)		1.36^§^ (0.97–1.91)		0.59**(0.48–0.73)	
	The 4th quantile, Q4		1.39 (0.55–3.53)		0.54 (0.25–1.14)		1.22 (0.60–2.49)		0.82 (0.51–1.32)	1.18 (0.69–2.01)		0.94 (0.69–1.28)		1.63** (1.18–2.25)		0.71** (0.58–0.87)	

*Note*: Multilevel logistic regression adjusted for survey year, age of the mother, education attainment, employment status, HIV knowledge, number of children under five in the household, regions with higher/lower migration rates, and residency.

^§^ *p* < 0.10

*p < 0.05

**p < 0.01

## Discussion

Sexual health is a key and integral part of the overall health of the population, and it is particularly important to women during pregnancy and motherhood as it is inextricably linked to the women’s reproductive health [34]. The study of sexual health is particularly relevant to specific groups of mothers that are in greater risks of incurring poor sexual health; these include low SES mothers and mothers who are married to a migrant [4, 35]. Differences in sexual health over time further add nuances to a study and help us to understand how periods of social transition influence sexual health. Nevertheless, there has been little research that has explored the sexual health of LBMs while taking into account their economic situation and changes over time. The present study used multilevel logistic regression models that not only strives to assess the discrepancies in sexual health between LBMs and non-LBMs in Armenia, but also examined how the differences in their SES status influenced their sexual health over time, namely from 2010 to 2015.

Our results indicated an incremental increase in adverse sexual health outcomes that particularly affected mothers from economically deprived households. Rates of self-reported STI symptoms and IPV increased over this period; in contrast, the rate of negotiating power over sex decreased among Armenian mothers. The rise in economic inequalities and the lack of economic opportunities among women during the studied period in Armenia thus should help us to understand these changes in sexual health over time. During the last decade the Armenian economy has shown remarkable improvements that have helped to reduce poverty and create more employment. However, this economic growth has been a double-edged sword. Along with this optimistic economic situation, economic inequalities have also arisen in Armenian society and the gap between the rich and the poor has magnified [26]. The Gini index, a broadly used indicator of economic inequality, has continued to increase from 2010 onwards in Armenia. In 2010 the Gini index was 30, and by 2015 it had increased to 32.6 and by 2017 it had risen to 33.6 [36]. Another downside of this economic growth is that female labor force participation did not improve during this period. According to data from the World Bank, the unemployment rate among Armenian women in 2010 was 21.3%, and this has remain fairly steady until 2015 when it stood at 19.3%; this is still higher than that of the total population, which stood at 18.3% [37]. If we compare labor force participation rate for females and males, the differences become more striking. In 2010 the female labor force participation rate was 52.2%, while that for males was 72.3% and this situation did not get improve over the period from 2010 to 2015. In 2015 the rate of female labor force participation had increased slightly to 54.16%, but it had dropped again to 52.76% by 2017. Over the whole period, male labor force participation rates had remained much higher at about 70% [38]. These inequalities in both the economic and the gender sphere are likely to have increased the economic hardships of mothers from the poorest households over this five year period [27, 39]. As a consequence, this situation is also likely to have strengthened the dependence of low SES mothers on their partner’s income [12], which would accentuate IPV [40] and increase the risk of acquiring STIs [41]. These findings are in line with Armenia's gender-based violence national data. In 2010 the percentage of women experiencing physical, psychological and sexual violence was 8.9%, 25.0% and 3.3%, respectively, while in 2016 these percentages had increased to 12.5%, 45.9% and 14.6%, respectively, [42, 43].

The results of the present study provide empirical findings that support the current consensus in the public health literature, namely that social disparities affect health [44]. Increased economic hardship among the poorest mothers is likely to have resulted in the observed rise in adverse sexual outcomes and may also explain the significant contribution of household wealth to the increment in self-reported STI symptoms. Our analysis shows that the effect of household wealth on STI symptoms intensified from 2010 to 2015, in particular among mothers from the first and second household wealth quintiles (the poorest and the poorer). For instance, the likelihood of Armenian mothers coming from the poorest households to report STIs in 2015 was almost three times higher than their counterparts in 2010 (1.06 x 2.53 = 2.7). Similarly, mothers from the poorer household wealth quintiles increased their likelihood of reporting STIs by 2.43 times over the period 2010–2015 (0.82 x 2.97 = 2.43). The present study also examined the contribution of left-behind status to these differences in sexual health over time, and no significant findings were obtained (results not shown). Overall, the above findings highlight not only the accentuated vulnerability of low economic status mothers to adverse sexual health outcomes, but also that this vulnerability has intensified in Armenia over the studied period.

As expected, among the whole sample of Armenian mothers, our analysis identified that household wealth had a significant influence on reporting STI symptoms, the utilization of HIV testing, the reporting of IPV, and whether an individual had negotiating power over sex. These findings are consistent with other studies that have reported that, coming from one of the highest household wealth quintiles, compared to the lowest quintile, has a protective effect in terms of IPV [19] and facilitates HIV testing services utilization [45]. This clearly reasserts that low SES women are more vulnerable to poor sexual health. What is more interesting is that higher household wealth has effects on sexual health that are different between LBMs and non-LBMs. Household wealth was associated with all of the non-LBMs sexual health outcomes, whilst the likelihood of LBMs reporting STIs symptoms was not affected by their economic position. Having a higher SES may improve the sexual health outcomes among non-LBMs, but any such effects are smaller among LBMs. This finding may reflect the fact that there are different mechanisms leading to LBMs being affected by poor sexual health than non-LBMs.

Our analysis has demonstrated that LBMs are more likely to report STIs than non-LBMs. The higher prevalence of HIV/STI among migrant husbands may place LBMs at higher risk of STIs regardless of their economic status. This finding is consistent with another study in Armenia that found that women who were married to a migrant reported more STI symptoms than women who were married to a non-migrant [7]. Furthermore, other studies in China [46], Nepal [47], and India [48] have highlighted the vulnerability of left-behind women to HIV/STIs. It is notable that there were no significant differences in HIV testing utilization between LBMs and non-LBMs that could be identified. This may reflect the successful governmental campaigns targeting Armenian mothers that have been aimed at increasing access to HIV testing services; these have had the aim of reducing mother-to-child transmission of HIV [49]. Nevertheless, our findings emphasize the specific risks associated with being a LBM, particularly regarding infection with an STI.

The present study has several limitations that should be acknowledged. This study does not include a number of important factors that would have helped us to understand the vulnerabilities of LBMs, such as the characteristics of their male partners and variables related to the social networks available to the LBMs. Furthermore, the use of questionnaires to assess sensitive issues such as STI symptoms and the acceptance to IPV could have led to information bias. For example, one obvious effect might be that these issues have been under-reported. However, it should be mentioned that LBMs are more prone to report STI than non-LBMS as they are more aware about the risky behaviors of their migrant partners. This study has relied on self-reporting of STI symptoms, and thus the diagnoses were not verified by a medical doctor. However, self-reporting is the standard method of collecting STI data. Acceptance of IPV was used as a proxy for direct violence in this study. A study has confirmed the association between acceptance of IPV and experiencing IPV [16] and experience during data collection has shown that attitudes are less prone to bias than behaviors. Finally, the cross-sectional design of this study makes it difficult to infer causal relationships. Notwithstanding the above limitations, the present study also has numerous strengths. These include the use of recent data from an extensive nationally representative survey, the application of adjusted models that include a wide range of potential confounders that will increase the internal validly of the study, and the performance of multilevel modeling strategies that should avoid possible bias due to sample clustering.

## Conclusion

The findings of this study contributed to a better understanding of the specific vulnerabilities that face LBMs regarding poor sexual health in context of the presence of remarkable economic inequalities; specifically, the analysis attempts to disentangle the dynamics of interplay between left-behind status, economic position and sexual health. The findings show that both economic position and left-behind migration status do independently influence the sexual health of Armenian mothers. This study also pointed out that adverse sexual health outcomes increased over the period 2010 to 2015 among Armenian mothers and that this particularly affected mothers from deprived households. These findings should encourage in the future the development of program strategies in Armenia that are socioeconomic-status and migration-status appropriate.
